# Improving drug safety in hospitals: a retrospective study on the potential of adverse drug events coded in routine data

**DOI:** 10.1186/s12913-019-4381-x

**Published:** 2019-08-08

**Authors:** Nils Kuklik, Jürgen Stausberg, Marjan Amiri, Karl-Heinz Jöckel

**Affiliations:** 1 Institute of Medical Informatics, Biometry and Epidemiology, University Hospital Essen, University of Duisburg-Essen, Hufelandstr. 55, 45147 Essen, Germany; 2 Centre for Clinical Trials Essen (ZKSE), University Hospital Essen, University of Duisburg-Essen, Essen, Germany

**Keywords:** Routine data, ICD-10, Adverse drug event, Medication error, Drug safety

## Abstract

**Background:**

Adverse drug events (ADEs) that occur during hospitalization are an ongoing medical concern. Systematic strategies for ADE identification are lacking. The aim of this study was to evaluate the potential to identify adverse drug events caused by medication errors (preventable ADEs, pADEs), and previously unknown adverse drug reactions (ADRs or non-preventable ADEs, npADEs) in inpatients by combining diagnosis codes in routine data with a chart review.

**Methods:**

Diagnoses of inpatients are routinely coded using the International Classification of Diseases, 10th Revision (ICD-10). A total of 2326 cases were sampled from routine data of four hospitals using a set of ICD-10 German Modification ADE codes. Following a chart review, cases were evaluated in a standardized process with regard to drug relation and preventability of events.

**Results:**

By chart review, 1302 cases were classified as hospital-acquired and included in the evaluation. This yielded 1285 cases indicating an ADE. 96.8% of ADEs (1244 ADEs) were classified as known npADEs, only three cases as suspected previously unknown npADEs, one case as event after drug abuse. A total of 37 ADEs were classified as preventable (2.9% of all ADEs) by identifying a medication error as probable cause. The prevalence of pADEs varied considerably between included ADE codes, with hemorrhagic diathesis due to coumarins and localized skin eruptions showing the highest rates (8.7 and 9.1%, respectively). Most frequent medication errors were non-compliance to a known allergy, and improper dose.

**Conclusions:**

When focusing on specific ADE codes, routine data can be used as markers for npADEs and medication errors, thus providing a meaningful complement to existing drug surveillance systems. However, the prevalence of medication errors is lower than in former studies on the frequency of pADEs.

## Background

Patients often experience adverse drug events (ADEs) during hospitalization [1, 2]. Such inpatient ADEs pose a considerable health and economic burden on the patients as well as on the health care system [3–5]. A significant number of inpatient ADEs are caused by medication errors and can be prevented (pADEs) [1, 6, 7]. By release of the action plans for improvement of medication safety by the Federal Ministry of Health in Germany, various measures have been implemented and promoted over the past decade in order to prevent and identify ADEs [8]. In developed countries, hospitals increasingly use clinical decision support systems and computerized physician order entry systems to reduce prescription errors [9, 10].

To further improve drug safety it is crucial to overcome the lack of systematic detection and reporting of non-preventable adverse drug events (npADEs) and pADEs, and to perform an ongoing root cause analysis in order to identify factors that contribute to errors in hospitals [11, 12]. Spontaneous reporting systems and critical incident reporting systems (CIRS) for reporting ADEs are internationally established, but they suffer from acceptance problems in daily routine [13]. Although the total number of spontaneous reports in Germany has been increasing for several years, the number is still low and the increase is mainly a result of higher reporting rates from pharmaceutical companies and patients [14].

Important data sources in hospitals are the diagnoses of inpatients routinely coded in Germany with the ICD-10 German Modification (ICD-10-GM) [15]. The codes are part of the hospital routine data, which are transmitted promptly to sickness funds and annually to the Institute for the Hospital Remuneration System as a standardized data set. Various studies have identified and validated ICD-10 codes as high-precision markers for the identification of ADEs (so-called ADE codes) [16–19]. It was further reported that 50% of inpatient ADEs are coded as disease in the routine data [19], including between 7 and 12% [18–20] that are coded as drug-related disease. Despite this moderate sensitivity, given the high precision and nationwide availability of ADE codes, routine data could complement existing pharmacovigilance systems and thereby contribute to the improvement of drug safety in hospitals.

Therefore, the aim of this study was to evaluate the potential of utilizing ADE codes encoded in routine data as a complementary drug safety source by identifying a) preventable ADEs including causes and contributing factors of medication errors, and b) previously unknown non-preventable ADEs, those that are not listed in the Summary of Product Characteristics (SmPC). The results of the study could stimulate the use of routine data as a pharmacovigilance resource.

## Methods

### Definitions

The following definitions are used allowing a clear distinction between non-preventable and preventable ADEs [21, 22]: an adverse drug event (ADE) is any harmful incident resulting from medical intervention related to a drug. ADEs are subdivided into non-preventable ADEs (npADEs) defined as harmful and unintended reactions to a drug after its appropriate use (adverse drug reaction), and preventable ADEs (pADEs), defined as harm to the patient due to errors in the drug treatment process. The definition of a npADE is consistent with the definition of an adverse reaction in ICD-10-GM, version 2018: adverse reaction of a drug that has been correctly prescribed and properly administered [15].

### Study design and database

We conducted a retrospective, multicenter, observational study with an explorative approach using secondary data analysis to identify preventable and non-preventable harm in inpatients. Hospital discharge data from four full-service, non-academic hospitals in Germany of the calendar years 2015 and 2016 were used. The hospitals are located in cities with a population between 45,000 and 180,000 and together operate 2300 beds and treat 109,000 inpatients. The routine data contain inpatient conditions coded by ICD-10-GM with one principal diagnosis and several additional diagnoses. The principal diagnosis is defined as “that condition established after study to be chiefly responsible for occasioning the admission of the patient to the hospital for care”, whereas additional diagnoses are defined as “all conditions that coexist at the time of the principal diagnosis, or that develop during the hospital stay” [15]. Since the focus of this study was on hospital-acquired ADEs (nosocomial conditions acquired during hospitalization) and as by definition hospital-acquired events cannot be assigned as principal diagnosis, only additional diagnoses were included. However, because additional diagnoses include comorbidities present at admission as well as hospital-acquired complications, and because the ICD-10-GM does not contain a Present on Admission (POA) indicator, the time of occurrence of corresponding events was determined during the chart review process.

### Sample selection

In previous studies of the authors, the general suitability of ICD-10 codes for ADE identification was investigated by calculating prevalence, precision and sensitivity of ICD-10 additional diagnosis codes [19, 23]. Based on these results, ADE codes were selected for evaluation in this study if they a) indicate predominantly hospital-acquired events, b) have been validated as codes representing ADEs with high precision (positive predictive values 68 to 94%, see [19]), and c) occurred more frequently compared to other ADE codes. One ADE code identifies one inpatient stay and is defined as one observational unit (hereinafter called “case”). In each hospital, all cases in each code group that fulfilled inclusion criteria were independently retrieved from the respective routine data, resulting in a sample of 2326 cases. A case was identified in the hospital information system by the patient identifier linked to the ICD-10 code representing the ADE. Then, the patient chart was retrieved either in electronic or in paper-based format. Table 1 shows the included codes and the number of cases.

**Table 1:** Included ICD-10-GM codes and number of cases

Code/Code group	Term	*N*
A04.7	Enterocolitis due to *Clostridium difficile*	362
D68.33; D68.34; D68.35	Hemorrhagic diathesis due to coumarins (vitamin K antagonists)/due to heparins/due to other anticoagulants	488
D69.52; D69.53	Secondary thrombocytopenia: Heparin induced thrombocytopenia type I/II	114
I95.2	Hypotension due to drugs	563
K52.1	Toxic gastroenteritis and colitis	155
L27.0; L27.1	Generalized/localized skin eruption due to drugs and medicaments	506
N99.0	Postprocedural renal failure	138
Total		2326

### Data extraction and evaluation

**Fig. 1:**
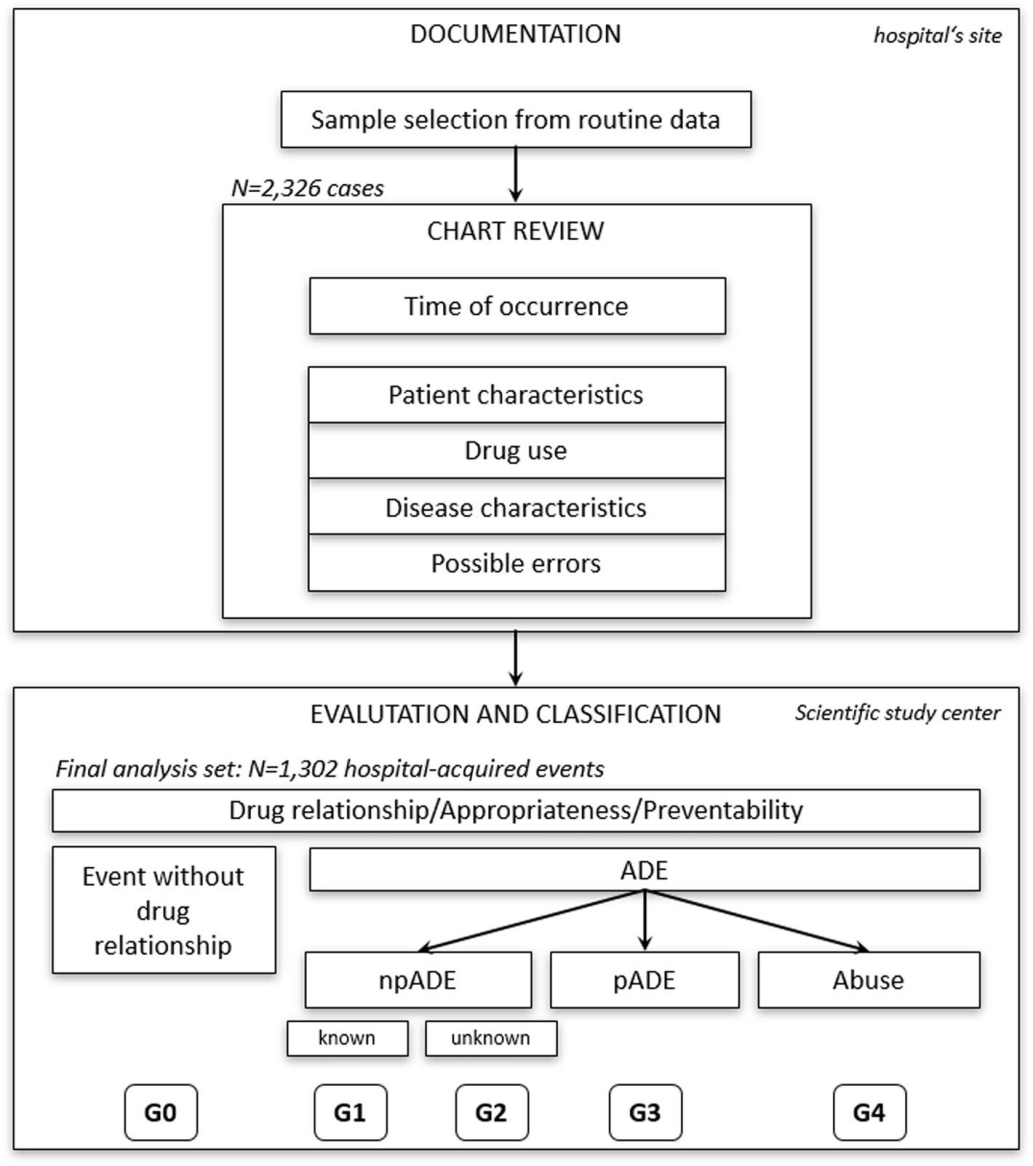
Schematic representation of the review and evaluation process. ADE = adverse drug event; npADE = non-preventable adverse drug event; pADE = preventable adverse drug event

Data for the analysis were recorded and evaluated in a multi-level, standardized procedure (Fig. 1). First, experienced personnel with medicinal and pharmaceutical background performed the chart review and documentation of event characteristics at the hospital’s site after completing a two-month study-specific training. To assure the data quality in the chart review process, multiple on-site visits were carried out. Starting point for a chart review was the diagnosis of one of the ADE codes listed in Table 1. After identification of the event in the medical record, data regarding the time point of the event, the relationship of the event to a drug if reported by the hospital staff, patient characteristics, drugs taken before event, patient’s known allergies and comorbidities, and source of information (physician or nurse) were extracted from the medical charts and the hospital information system. Data were recorded on a standardized data collection form. The parameters to be collected to identify and characterize a medication error were adopted from the taxonomy of medication errors from the National Coordinating Council for Medication Error Reporting and Prevention (NCC MERP, USA) [24].

At the scientific study center, the completed data collection forms were evaluated. All cases that occurred during hospitalization were classified according to type of adverse event. The underlying root cause of the occurrence of the event was assessed and related drugs to the ADE were assigned by taking into account the drug relationships recorded by the hospitals and by inspecting the SmPCs of administered drugs. A preventability assessment of the ADEs was performed to distinguish pADEs from npADEs by considering errors recorded by the hospital staff and by comparing collected data with the SmPC’s application instructions. A clinical decision support system (Software ID DIACOS PHARMA; ID Information und Dokumentation im Gesundheitswesen GmbH, Berlin) was used to support the assessment process. The following groups were defined to categorize the ADEs.

- **G0 – event without drug relationship:** Adverse event for which no related drug therapy was identified.
- **G1 – known non-preventable ADE:** ADE after proper use without indication of a medication error; ADE listed in the SmPC.
- **G2 – suspected previously unknown non-preventable ADE:** ADE after proper use without indication of a medication error; ADE not listed in the SmPC.
- **G3 – preventable ADE:** ADE with medication error as probable cause for reported event.
- **G4 – drug abuse:** ADE because of drug abuse by the patient.

No personnel of the study center was involved in the data extraction process at the hospital’s site. At the scientific study center, cases were evaluated and categorized by author MA. All assessments were reviewed by author NK. For cases assigned to G2 and G3, a final consensus agreement was achieved by authors JS and NK. Absolute and relative frequencies and exact 95% confidence intervals (CI) were calculated using SAS (SAS Institute Inc., Release 9.4).

## Results

By chart review, data from 2326 cases were extracted. Of the reviewed cases, 1328 cases encoded events that occurred during hospitalization (57.1% of 2326), 747 cases represented events present at admission, and 251 with unknown onset date. Overall, 26 cases were excluded from the set of hospital-acquired events due to incomplete data or implausibility. Therefore, 1302 cases were included in the final analysis and assigned to groups G0 - G4 (Table 2).

**Table 2:** Classification of ADEs per ADE code: absolute and relative frequencies

Group	N[%] cases per ADE code													
	A04.7	D68.33	D68.34	D68.35	D69.52	D69.53	I95.2	K52.1	L27.0	L27.1	N99.0		Total	95% CI
G0 - event without drug relationship	15 [7.1]	0	0	0	0	0	0	0	0	0	2 [1.9]		17 [1.3]	0.8–2.1
G1 - known npADE	195 [92.9]	42 [91.3]	7 [100]	12 [100]	9 [100]	41 [100]	401 [99.0]	87 [98.9]	209 [93.7]	138 [89.6]	103 [96.2]		1244 [95.6]	94.3–96.6
G2 – previously unknown npADE	0	0	0	0	0	0	0	1 [1.1]	0	2 [1.3]	0		3 [0.2]	0.1–0.7
G3 - pADE	0	4 [8.7]	0	0	0	0	3 [0.7]	0	14 [6.3]	14 [9.1]	2 [1.9]		37 [2.8]	2.0–3.9
G4 – event after drug abuse	0	0	0	0	0	0	1 [0.3]	0	0	0	0		1 [0.1]	0–0.4
Total	210	46	7	12	9	41	405	88	223	154	107		1302	

*npADE* non-preventable adverse drug event, *pADE* preventable adverse drug event, *CI* confidence interval

Most of the cases were confirmed as ADE (G1-G4). A small percentage of the codes enterocolitis due to Clostridium difficile (A04.7-), and postprocedural renal failure (N99.0) represented events without drug relationship (G0). While 90 to 100% of cases across all codes were classified as known npADEs, only three cases were classified as suspected previously unknown npADEs, i.e. the ADE was not listed in the SmPC: Eliquis (active ingredient: Apixaban) associated with toxic gastroenteritis and colitis (K52.1), and Valoron (active ingredient: Tilidine; two cases) associated with localized skin eruption (L27.1).

A total of 37 cases (2.9% of all 1285 ADEs) represented pADEs. pADEs were identified in association with the ADE codes hemorrhagic diathesis due to coumarins (D68.33), hypotension due to drugs (I95.2), generalized and localized skin eruption (L27.0 and L27.1), and postprocedural renal failure (N99.0). Among pADEs, the codes D68.33, L27.0, and L27.1 showed the highest rates. One case with the ADE code hypotension due to drugs (I95.2) was related to drug abuse by the patient.

Out of the 37 cases with pADEs, 28 medication errors were related to skin eruptions. The non-compliance to a known allergy (27 cases) was the most frequent type of medication error (Table 3). Of these, 24 cases were associated with allergies to antibiotics. Improper dosing was rarely observed (seven cases). Possible causes and contributing factors could only be identified for a small proportion of medication errors.

**Table 3:** Types, causes and factors of medication errors

ADE code	Type of medication error	*N*	Causes/Factors	*N*
D68.33	Wrong time of administration	1	–	
	Improper dose	3	Heavy workload	1
I95.2	Improper dose	3	Verbal miscommunication	1
L27.0	Contraindication, known allergy (antibiotic)	14	Transcription error Written miscommunication	2 1
L27.1	Contraindication, known allergy (antibiotic)	10	Transcription error Written miscommunication	2 1
	Contraindication, known allergy (analgesic)	2	Verbal miscommunication	1
	Contraindication, known allergy (heparin)	1	–	
	Improper dose	1	–	
N99.0	Contraindication, comorbidity	1	–	
	Contraindication, drug-drug interaction	1	–	
	Total	37		

*ADE* adverse drug event

## Discussion

In our study, codes of the ICD-10-GM (ADE codes) were analyzed to assess their potential for the detection of pADEs and previously unknown npADEs. As observed in the preceding validation study [19], the selected ADE codes represented high-precision markers for drug-related conditions that, with the exception of hemorrhagic diatheses, by the majority developed during hospitalization. These codes are thus suitable for the analysis of hospital-acquired ADEs.

The evaluation of the ADE codes revealed no evidence of medication errors in the vast majority of cases. Only 2.9% of all ADEs (G1-G4) were classified as probable consequences of medication errors and therefore as preventable (pADEs). However, the prevalence of pADEs varied significantly between ADE codes, ranging from 0 to 9.1%. In particular, higher rates were found for the ADE codes hemorrhagic diathesis associated with administration of vitamin K antagonists (8.7%), and skin eruptions (9.1%), mostly due to antibiotics. Both drug groups are frequently reported in association with hospital-acquired medication errors [7, 25, 26]. Former studies found higher rates of hospital-acquired pADEs compared to the results presented. For example, a prospective study at two hospitals in the Netherlands reported a rate of 5% inpatient pADEs [22], a prospective study in the UK found a pADE rate of 52%, and classified 47% of them as “possible” and 5% as “definite” [25]. One meta-analysis reviewing eight prospective studies from the years 1994–2010 [6] assessed 45% of all hospital-acquired ADEs to be preventable, whereas another meta-analysis evaluating nine prospective and retrospective studies from the years 2006 to 2014 [7] reported 32% pADEs. However, differences in methodology and study population complicate the comparison of the results. A continuous improvement of quality standards in the drug therapy process and a more frequent use of electronic systems contribute to a reduction of preventable adverse events [9, 10]. This might explain the rarity of pADEs determined in this study, indicating a possible overestimation of the burden of medication errors in the current discussion on drug safety in the inpatient setting. However, considering the total number of inpatients in Germany and high percentage of ADE codes, rates of pADEs as determined in this study still demonstrate the ongoing relevance of drug safety improvement.

Possible causes and contributing factors of medication errors could only be determined in a few cases. Hospital staff related human factors such as heavy workload, transmission errors between documents, and communication deficits could be identified. To increase the patient’s safety, a systematic root cause analysis of medication errors at hospitals is essential in order to identify conditions in which medication errors are favored, to initiate structural changes to remedy them, and to define and optimize specific workflows. These measures have received increasing attention in recent years, for example through implementation of CIRS [27] or the formulation of standard operating procedures [28]. In total, three cases of suspected previously unknown npADEs were identified. The low number and lack of information on the actual frequency of previously unknown ADEs in hospitals hampers a final qualitative assessment of the usability of routine data in this context. Therefore, the potential of routine data for the detection of previously unknown npADEs cannot be conclusively derived. A validation of the prevalence having a larger sample is recommended.

Limitations in the interpretation of the presented results can be discussed at different levels. Generally, routine data have only a moderate sensitivity for inpatient ADEs. As reported in the preceding validation study, 50% of hospital-acquired ADEs were coded as disease in the routine data, from which a subgroup of 12% was coded as drug-associated disease [19]. A possible impact of under-reporting of ADEs in routine data on the rate of pADEs was not verified in this study. It can be argued that clinical personnel may be reluctant to code events related to medication errors and that there is a lack of information in the source data. On the other hand, this effect may be compensated because the severity and relevance of pADEs may in turn lead to higher coding rates. Therefore, taking into account the impact of under-reporting of pADEs but also the tendency to code ADEs with high severity more frequently, there is no evidence that the sensitivity of ADE codes indicating medication errors is lower than of ADE codes encoding non-preventable ADEs. The suspected medication errors and previously unknown npADEs identified in this work are distributed over a small set of ADE codes. Although the most frequent ADE codes were included in the analysis, it is not easily possible to generalize the prevalence rates determined in this study to other codes. The hospitals in this study have no specific characteristics. The evaluation based on nationwide uniform ICD-10 codes that are coded according to standardized guidelines [15]. Therefore, a generalization of the results to other hospitals in Germany is reasonable. However, due to possible structural differences in different countries with regard to the pharmacovigilance infrastructure, a generalizability to other countries is only possible to a limited extent. Data on the frequencies of additional diagnoses in Germany show that unspecific codes are regularly used to code events [23]. This includes codes such as T78.4 “Other and unspecified allergy” and T88.7 “Unspecified adverse effect of drug or medicament” - codes which do not directly identify the underlying event and which were therefore excluded from the study. Further studies are necessary to validate the impact of these codes on the rate of hospital-acquired pADEs.

## Conclusion

Detection of pADEs and previously unknown npADEs in everyday clinical practice is a major challenge in healthcare. Our study confirmed the potential of utilizing ADE codes encoded in routine data as a complementary drug safety source. Furthermore, our data indicated that pADEs occur less frequently than expected. The majority of npADEs were mentioned in the SmPCs of related drugs.

The Drug Commission of the German Medical Association is currently developing a reporting system to systematically collect and evaluate medication errors within the framework of the spontaneous reporting system for ADRs [29]. To address the under-reporting of ADEs, additional strategies to collect drug safety data are needed. Having a comprehensive and standardized acquisition, routine data can be effectively used as a complementary data source to detect medication errors. Our results demonstrate that the majority of ADEs coded in routine data are known npADEs. However, using routine data as markers for pADEs in combination with chart review is reasonable when focusing on specific ICD-10 codes. In a study from South Korea, ADE codes from nationwide routine data have been used as a basis to evaluate drug safety following the realization of an electronic drug prescription system [30]. Furthermore, pADEs coded in routine data can provide important information for systematic prospective quality assessments in hospitals in order to implement preventive, risk-reducing measures in hospital management. One important step towards greater use of routine data in drug safety is the identification of further, suitable ADE codes [31]. The implementation of a POA indicator in the German version of the ICD-10, a more strict specification of medication error coding in routine data, and not least raising awareness of ADE coding in hospitals can further increase the potential of routine data within the framework of drug safety.

## Data Availability

The data that support the findings of this study are available from the participating hospitals but restrictions apply to the availability of these data, which were used under license for the current study, and so are not publicly available. Data are however available from the authors upon reasonable request and if no interests of the participating hospitals are affected.

## Abbreviations

ADE: Adverse Drug Event
ADR: Adverse Drug Reaction
CIRS: Critical Incident Reporting System
ICD-10-GM: International Classification of Diseases, 10th Revision, German Modification
npADE: non-preventable Adverse Drug Event
pADE: preventable Adverse Drug Event
POA: Present on Admission
SmPC: Summary of Product Characteristics
